# Machine Learning and Multi-Omics Integration to Reveal Biomarkers and Microbial Community Assembly Differences in Abnormal Stacking Fermentation of Sauce-Flavor *Baijiu*

**DOI:** 10.3390/foods14020245

**Published:** 2025-01-14

**Authors:** Shuai Li, Yueran Han, Ming Yan, Shuyi Qiu, Jun Lu

**Affiliations:** 1 College of Liquor and Food Engineering, Key Laboratory of Fermentation Engineering and Biological Pharmacy of Guizhou Province, Guizhou University, Guiyang 550025, China; lishuai2057@163.com; 2 Guizhou Guotai Distillery Co., Ltd., Renhuai 564501, China; hanyueran1210@163.com (Y.H.); yanming554@163.com (M.Y.)

**Keywords:** machine learning, multi-omics, community assembly, biomarker, abnormal fermentation

## Abstract

Stacking fermentation is critical in sauce-flavor *Baijiu* production, but winter production often sees abnormal fermentations, like Waistline and Sub-Temp fermentation, affecting yield and quality. This study used three machine learning models (Logistic Regression, KNN, and Random Forest) combined with multi-omics (metagenomics and flavoromics) to develop a classification model for abnormal fermentation. SHAP analysis identified 13 Sub-Temp Fermentation and 9 Waistline microbial biomarkers, along with 9 Sub-Temp Fermentation and 12 Waistline flavor biomarkers. *Komagataeibacter* and *Gluconacetobacter* are key for normal fermentation, while *Ligilactobacillus* and *Lactobacillus* are critical in abnormal cases. Excessive acid and ester markers caused unbalanced aromas in abnormal fermentations. Additionally, ecological models reveal the bacterial community assembly in abnormal fermentations was influenced by stochastic factors, while the fungal community assembly was influenced by deterministic factors. RDA analysis shows that moisture significantly drove Sub-Temp fermentation. Differential gene analysis and KEGG pathway enrichment identify metabolic pathways for flavor markers. This study provides a theoretical basis for regulating stacking fermentation and ensuring *Baijiu* quality.

## 1. Introduction

Chinese *Baijiu* is made from grains through saccharification, fermentation, distillation, and blending [1]. It is considered one of the world’s six major distilled spirits, along with brandy, whiskey, vodka, gin, and rum [2]. Sauce-flavor *Baijiu*, a typical representative of Chinese *Baijiu*, owes its unique flavor and quality to its complex and multi-step traditional brewing process [3].

Stacking fermentation is the most critical process in the traditional brewing system of sauce-flavor *Baijiu* [4]. It typically involves steaming and gelatinizing the raw materials, cooling and adding Daqu (a saccharifying and fermenting agent made from grains), and then stacking them in a tent-like shape in an open brewing environment [5]. This process enriches essential brewing microorganisms from the environment and produces important sauce-flavor compounds and flavor precursors [6]. Previous studies have shown that *Jiupei* (fermented grains) without stacking fermentation has significantly lower levels of various aromatic compounds, resulting in less prominent sauce-flavor characteristics in the *Baijiu* [4]. However, stacking fermentation often faces challenges, such as climate instability or improper manual operations, leading to microbial dysbiosis or metabolic disorders in *Jiupei*. This results in clumping and moldiness, dark coloration, and slow temperature rise, phenomena we defined as “Waistline” when white colonies and clumping occur in the middle section of stacking fermentation and “Sub-Temp Fermentation” for overall slow temperature rise during the fermentation process. Currently, the identification of abnormal stacking fermentation phenomena relies solely on subjective sensory experience, lacking scientific and objective evaluation standards. Additionally, in the open and complex stacking fermentation ecosystem, taxa within the community occupy optimal ecological niches and assemble microbial communities based on ecological phenomena such as priority effects [7,8,9]. However, the significant environmental heterogeneity in different stacking fermentation states, the ecological niches of microbial species, and the contributions of stochastic and deterministic ecological processes to microbial community assembly in different fermentation states remain unclear. Therefore, the industry urgently needs to identify key biomarkers causing abnormal stacking fermentation based on microbial and metabolic levels and understand the differences in microbial community composition and assembly mechanisms that affect abnormal stacking fermentation. This will provide scientific theoretical guidance for production, allowing the timely regulation and improvement of fermentation levels to ensure the quality of *Baijiu*.

In recent years, omics technologies and artificial intelligence have been continuously updated [10]. Multi-omics technologies are increasingly emerging in the traditional fermented food industry [11]. Yang et al. used multi-omics technologies to elucidate that differences in microbial ecological niches in different spatial environments are a significant reason for the micro-ecological differentiation of high-temperature Daqu in sauce-flavor *Baijiu* [12]. Zhang et al. applied flavoromics techniques combined with comprehensive 2D gas chromatography coupled with mass spectrometry to elucidate key volatile flavor compounds in beer and wine [13]. However, the heterogeneity in sample types and scales between different omics data increases the difficulty of data integration [14]. Additionally, omics technologies often contain [1] a large number of missing values and noise, requiring higher-level algorithms for preprocessing [15]. To address the issues in omics technologies, machine learning holds great potential in data processing and analysis [16]. By optimizing algorithms and iteratively training models, it can extract and reveal functionally significant relationships and biological mechanisms from complex multi-dimensional omics data, which are important for biological research and applications [17,18].

Based on the abnormal stacking fermentation phenomena, this study aimed to use multi-omics techniques such as metagenomics and flavoromics (non-targeted volatile flavor synthesis) to analyze the differences in microbial composition and volatile flavor compounds during abnormal stacking fermentation. By applying the neutral community model combined with the ecological null model, we aimed to characterize the differences in microbial community assembly mechanisms under different fermentation states. We selected three machine learning methods—Random Forests, Logistic Regression, and K-Nearest Neighbor (KNN)—to clarify the microbial and flavor markers causing abnormal stacking fermentation. By screening biomarkers and combining them with the KEGG database, we aimed to identify the key metabolic pathways responsible for abnormal stacking fermentation and elucidate the mechanisms of abnormal fermentation formation. This study provides scientific theoretical guidance for regulating and improving the stacking fermentation level of sauce-flavor *Baijiu* and ensuring its quality.

## 2. Materials and Methods

### 2.1. Sample Collection

The samples were obtained from Guizhou Guotai Distillery Co., Ltd., located in Maotai Town, Guizhou, China. The samples were *Jiupei* from the first round of stacking fermentation during the winter production of sauce-flavor *Baijiu* from three different batches covering the 1–7 day fermentation period. Following the sampling method of Wang et al. [6], we sampled the common abnormal fermentation phenomena in winter stacking fermentation, as shown in Figure S1a, as well as normal fermentation for comparison. The common fermentation states in stacking fermentation—normal fermentation, Waistline, and Sub-Temp Fermentation—were named NF, WL, and STF, respectively. Waistline *Jiupei* is milky white and blocky, with a slightly sour and astringent taste and a strong alcoholic aroma (Figure S1b). Normal fermentation *Jiupei* is bright in color and moderately loose, with a sweet liquor aroma and floral and fruity notes (Figure S1c). Sub-Temp Fermentation *Jiupei* is dark in color and loose, with a pungent sour and astringent taste (Figure S1d). The samples from the three treatment groups were from the same round of fermentation, with samples collected daily over the 1–7 day period. A total of 63 samples were collected (three replicates each): 21 normal fermentation samples, 21 Waistline samples, and 21 Sub-Temp Fermentation samples. Samples for physicochemical and volatile compound analysis were stored at 4 °C, while samples for metagenomic sequencing were stored at −80 °C.

### 2.2. Physicochemical Analysis

To characterize the physicochemical differences in *Jiupei* under different stacking fermentation states, we measured temperature, moisture, titratable acidity, reducing sugars, and starch content following the methods reported by Yang et al. [19]. Lactic acid and ethanol contents were measured using an M-100 biosensor (Siemens Technology, Beijing, China). The details are as follows: weigh 5.0 g of Jiupei and place it in a 250 mL beaker. Add 100 mL of water, then stir with a glass rod and let it soak for 30 min, stirring every 15 min. After soaking, adjust the pH to neutral or slightly acidic (pH 5–8) using a 20% NaOH solution. Let it stand for 10 min, then collect the supernatant for centrifugation. Use a desktop high-speed centrifuge and set the parameters for 5 min at 10,000 rpm. After centrifugation, collect the supernatant and proceed with testing using the M-100 biosensor.

### 2.3. Metagenomic Sequencing

Metagenomic sequencing of the *Jiupei* samples was performed by Personal Biotechnology Co., Ltd. (Shanghai, China). DNA was extracted from the samples using the OMEGA Mag-Bind Soil DNA Kit (M5635-02) from Omega Bio-Tek (Norcross, GA, USA). All sequencing analyses were conducted on the Illumina NovaSeq 6000 platform, following the methods outlined in the literature [20].

### 2.4. Identification of Functional Genes and Metabolic Pathways

Following the methods reported in the literature [21], MEGAHIT was used to construct a non-redundant gene set with 95% identity and 90% contrast coverage using default parameters, while Prodigal was used for each allele and scaffold. Functional annotation was performed using HUMAnN3 (v3.6), with annotation information and relative abundance tables obtained from KEGG based on Uniref 90 IDs and the Kyoto Encyclopedia of Genes and Genomes (KEGG) database.

### 2.5. Analysis of Volatile Compounds by HS-SPME-GC-MS

Following previously reported methods with slight modifications [22], 5.0 g of *Jiupei* was added to 20 mL of distilled water, ultrasonicated at 0 °C for 30 min, and then centrifuged at 8000× *g* for 5 min at 4 °C to obtain the supernatant. Next, 5 mL of the supernatant was added to a 20 mL headspace vial containing 2.0 g NaCl and 0.008 mL 2-octanol (internal standard, 100 mg/L). Volatile compounds were analyzed by HS-SPME-GC-MS (GC 7890N and MS 5975; Agilent Technologies, Santa Clara, CA, USA) using a DB-Wax column (30 m × 0.25 mm i.d., 0.25 μm film thickness; J&W Scientific, Folsom, CA, USA) with helium as the carrier gas at a constant flow rate of 2 mL/min. The injector temperature was maintained at 250 °C. The oven temperature was held at 50 °C for 2 min, then increased at a rate of 6 °C/min to 230 °C, and finally held at 230 °C for 20 min. Mass spectra were recorded in electron ionization mode (MS-EI) with an ionization energy of 70 eV and an ion source temperature of 230 °C. Full-scan acquisition was performed over a mass range of 30–350 amu. The retention index (RI) was calculated using a series of standard alkanes C5-C40 [22] as an external reference under the same conditions. The volatile compounds were qualitatively identified by comparing the mass spectra and RI values in the NIST17 standard library with a matching index ≥70% [22]. The semi-quantification of volatile compounds was calculated from peak areas according to the internal standard [13].

### 2.6. Mechanisms of Microbial Community Assembly

Based on the above genus-level microbial data, we constructed networks following the methods reported by Liu et al. [23]. We selected bacteria and fungi present in more than 75% of the samples and species with a relative abundance greater than 0.01% to construct the networks. The Spearman correlation matrix between genera was calculated based on the relative abundance of each sample. The random matrix theory (RMT) was used to automatically identify the appropriate similarity threshold before network construction. The network topological properties were characterized using the “igraph”, “psych”, and “Hmisc” packages in R (v.4.2.3), and the network graph was plotted using Gephi (version 0.9.5). The neutral community model (NCM) was applied to estimate the impact of stochastic processes on microbial community composition [24]. The NCM was constructed using the “Hmisc”, “minpack.lm”, and “stats4” packages in R (4.3.2), with the R^2^ value indicating the goodness of fit of the model. According to previous studies, the closer the R^2^ value is to 1, the greater the influence of stochastic processes on community assembly [25]. The niche width was calculated using the niche width function in the “spaa” package in R (4.3.2). The checkerboard score (C-score) null model method was applied to assess community species co-occurrence patterns and ecological stochasticity. The C-score and standardized effect size (SES) in the null model analysis were calculated using the “EcoSimR” package in R (4.3.2). According to previous studies [26], the magnitude of SES is interpreted as the strength of deterministic processes affecting microbial community composition, with larger SES values indicating a stronger influence of deterministic factors on community assembly [27].

### 2.7. Machine Learning Model Construction

Based on the machine learning model construction process shown in Figure S2, we screened for microorganisms and volatile flavor biomarkers causing abnormal fermentation. Data preprocessing involved applying the np.log1p (features) function in Python for natural log transformation, followed by standardization using the “StandardScaler” function. The dataset was split into training and testing sets in a 60:40 ratio using the train_test_split function, with stratified sampling (stratify = labels) to maintain consistent class distribution. Additionally, 80% of the training data was used for model training, while the remaining 20% of the training set was held out as a validation set. This ensures that hyperparameter tuning and model selection are performed on data that the model has not seen during training, helping to prevent overfitting [28]. Three machine learning models were selected: Random Forest, Logistic Regression, and KNN. Random Forest is an ensemble learning method that improves the accuracy and robustness of the model by combining the predictions of multiple decision trees. It uses randomness to reduce overfitting and is suitable for handling high-dimensional data. Logistic Regression is a linear classification model that maps the linear combination of features to a probability between 0 and 1 using the Sigmoid function, making it suitable for binary classification tasks. KNN classifies or regresses by calculating the distances between samples. As a non-parametric method, it makes no assumptions about the data distribution and directly predicts based on the training data. The specific setup parameters are as follows for the Random Forest model: we reported important parameters such as n_estimators = 100 (number of trees), max_depth = 10 (maximum depth of the tree), min_samples_split = 2 (minimum number of samples required for splitting), min_samples_leaf = 1 (minimum number of samples for leaf nodes), and max_features = “sqrt” (square root of the number of features considered for each split). Additionally, 42 features were selected for the Random Forest model, which was based on feature selection techniques to retain the most relevant features for classification. For the Logistic Regression model, we further added parameters such as penalty = “l2” (regularization type) and C = 0.001 (regularization strength). For the KNN model, we added n_neighbors = 5 and explicitly specified the distance metric as “Euclidean distance”. The SMOTE (Synthetic Minority Over-sampling Technique) was used to oversample the training set, ensuring data balance for model training and prediction. The models were evaluated using the AUC, F1-Score, Accuracy, Recall, and Precision [16]. Additionally, following previous methods [29], we employed the SHAP algorithm for global interpretation and calculated Shapley values for each feature across the models to rank feature importance, enhancing the credibility and transparency of the machine learning models. All machine learning models were implemented using the Scikit-learn library in Python 3.8 on the Jupyter Notebook platform, and the SHAP values were computed using the SHAP library.

### 2.8. Statistical Analysis and Visualization

All data statistical analyses were repeated 3 times. Statistical difference analysis was conducted using one-way analysis of variance (ANOVA) and T-tests in IBM SPSS Statistics (version 25.0; IBM Co., New York, NY, USA), with an adjusted *p*-value < 0.05 as the significance threshold. Principal component analysis (PCA) was performed using the “FactoMine” package in R (4.3.2), combined with permutational multivariate analysis of variance (Permanova) using the adonis function from the “vegan” package to determine significant differences in microbial communities and volatile compositions across different fermentation states. Dynamic changes in the microbial and flavor heatmap contents were visualized using the “heatmap” package in R (4.3.2). Based on Spearman’s rank correlation, the correlations between microorganisms and volatile compounds were calculated using the “psych” and “reshape2” packages in R (4.3.2). Significant correlations with *p* < 0.05 and |ρ| > 0.6 were considered valid [12]. Correlation networks were visualized using Gephi. Redundancy analysis (RDA) was conducted using the “capscale” function from the “vegan” package in R (4.3.2) to assess the correlations between microbial communities and various parameters. The differential fold change (FC) of microbial and flavor biomarkers and functional genes was calculated using the “DESeq2” package in R (4.3.2). A *p*-value < 0.05 and |log_2_FC| > 1 were considered significant [20]. Other statistical analyses and plotting were performed using OriginPro2023 (version 2023, OriginLab Corporation, Northampton, MA, USA), RStudio (v.4.3.2), and Adobe Illustrator CC2018 (version 2018, Adobe Systems Incorporated, Atlanta, GA, USA).

## 3. Results and Discussion

### 3.1. Analysis of Physicochemical Index Differences

**Figure 1:**
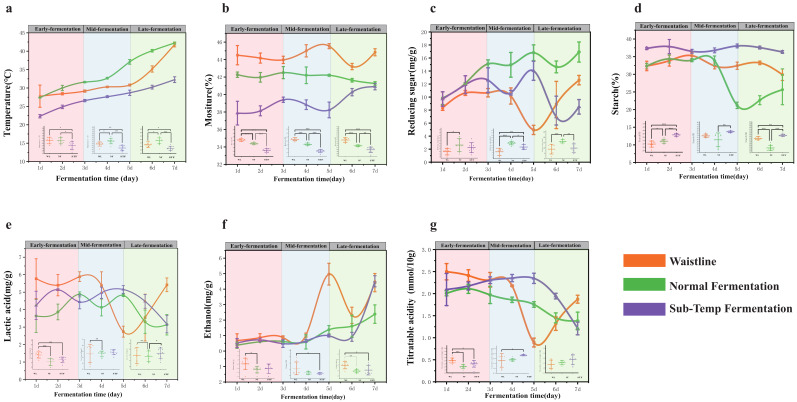
Dynamics of physicochemical indicators during stacking fermentation: (**a**) temperature, (**b**) moisture, (**c**) reducing sugar, (**d**) starch, (**e**) lactic acid, (**f**) ethanol, and (**g**) titratable acidity. The *, **, and *** indicate statistical significance at *p* < 0.05, *p* < 0.01, and *p* < 0.001, respectively.

During fermentation, physicochemical indicators are important driving factors for microbial community succession [19]. Seven physicochemical indicators showed significant differences between normal fermentation and the two types of abnormal fermentation (*p* < 0.05) (Figure S3). During the 1–7 day fermentation period, the temperature of normal fermentation was significantly higher than that of the two types of abnormal fermentation (*p* < 0.05) (Figure 1a). The average temperature of Sub-Temp Fermentation samples was only 25.6 ± 3.4 °C, not meeting the high-temperature fermentation standard for sauce-flavor *Baijiu* [4]. Additionally, the average moisture content during the 1–7 day fermentation period was also significantly lower in Sub-Temp Fermentation compared to normal fermentation (WL: 44.85 ± 0.8% > NF: 41.88 ± 0.5% > STF: 38.67 ± 1.2%) (*p* < 0.05) (Figure 1b). This indicated that during Sub-Temp Fermentation, microbial growth, reproduction, and metabolic activities were weaker, leading to poor bio-heat accumulation and low fermentation temperatures [30]. The average reducing sugar content varied significantly under different fermentation states (*p* < 0.05) (NF: 14.33 ± 2.6 mg/g > STF: 10.61 ± 2.8 mg/g > WL: 9.46 ± 3.1 mg/g) (Figure S3f). Especially in the mid-to-late fermentation stages, there were significant fluctuations in the reducing sugar content of the two types of abnormal fermentation. The reducing sugar content in Waistline samples dropped to a minimum of 4.88 mg/g at 3–5 days, then rapidly increased to 13.56 mg/g at 5–7 days. For Sub-Temp samples, the reducing sugar content peaked at 12.84 mg/g at 3–5 days, then rapidly decreased to 8.33 mg/g at 5–7 days (Figure 1c). These fluctuations reflected abnormal saccharification and fermentation rates in *Jiupei* during the fermentation process. Compared to abnormal fermentation, the hydrolysis of starch by amylase to produce reducing sugars was more intense in normal fermentation, indicating vigorous microbial metabolism. In contrast, the average starch content changes in the two types of incomplete abnormal fermentation were more stable and significantly higher than in normal fermentation (STF: 37.19 ± 0.97% > WL: 32.13 ± 1.86% > NF: 29.18 ± 5.7%) (Figure 1d and Figure S3g), indicating abnormal metabolic regulation during fermentation. Additionally, during the 1–7 day fermentation period, the average contents of lactic acid (NF: 3.96 ± 0.7 mg/g < STF: 4.51 ± 0.7 mg/g < WL: 4.87 ± 1.3 mg/g) (Figure S3d) and ethanol (NF: 1.08 ± 0.7 mg/g < STF: 1.25 ± 1.3 mg/g < WL: 2.16 ± 1.7 mg/g) (Figure S3e) were significantly lower in abnormal fermentation (*p* < 0.05), consistent with previous reports of high-acid, high-ethanol stress conditions.

### 3.2. Differences in Microbial Community Composition

**Figure 2:**
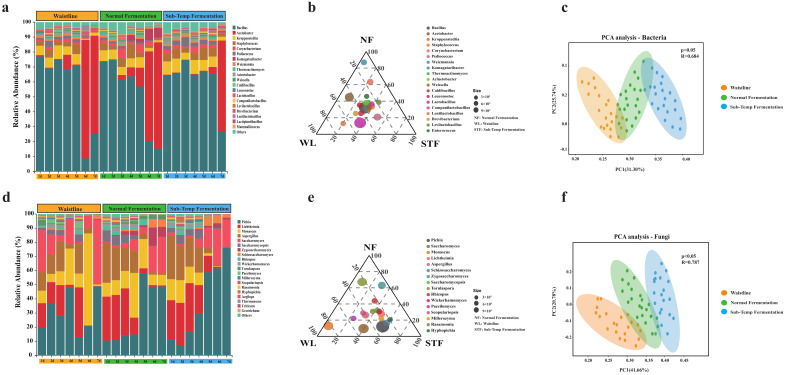
Microbial community dynamics during the fermentation process: (**a**) bacterial distribution at the genus−level of microbiota and (**d**) fungal distribution at the genus−level of microbiota. (**b**) Ternary phase diagram of dominant bacteria. (**e**) Ternary phase diagram of dominant fungi. (**c**) Score plot of bacterial compositional structure based on principal component analysis. (**f**) Score plot of fungal compositional structure based on principal component analysis.

The microbial community composition differences in different stacking fermentation states through metagenomic sequencing were characterized. After quality control, a total of 678.11 Gbps of clean data was obtained, followed by de novo assembly of the sequences. In the clean data from 63 samples, the proportion of effective sequence bases was 98.41%. A total of 475 bacterial genera and 135 fungal genera were detected in samples from the three different stacking fermentation states. Significant differences were found in the bacterial and fungal compositions between different fermentation states (*p* < 0.05), with the principal component analysis explaining 57.04% and 62.44% of the variance, respectively (Figure 2c,f).

The dominant bacterial genera (relative abundance > 1%) in different fermentation states included *Bacillus*, *Acetobacter*, *Kroppenstedtia*, *Staphylococcus*, *Corynebacterium*, *Pediococcus*, *Komagataeibacter*, *Weizmannia*, and *Thermoactinomyces* (Figure 2a), which is consistent with previous research results [19]. Among them, *Bacillus* (NF: 52.45% < WL: 56.34% < STF: 61.56%) and *Acetobacter* (WL: 22.34% > NF: 19.77% > STF: 9.56%) had an average relative abundance exceeding 60% throughout the 1–7 day fermentation period, making them the dominant bacterial genera in the stacking fermentation of sauce-flavor *Baijiu*. Previous studies have shown that certain Bacillus in the fermentation process of sauce-flavor *Baijiu* has the characteristic of producing a high yield of a pickle-like odor [31], which may be one reason why *Baijiu* from abnormal fermentation has a strong pickle-like odor. Additionally, *Komagataeibacter*, which improves the ecological stability and function of microbial communities in traditional fermentation [32], had a higher proportion in normal fermentation (Figure 2b).

The dominant fungal genera (relative abundance > 1%) in different fermentation states included *Pichia*, *Lichtheimia*, *Monascus*, *Aspergillus*, *Saccharomyces*, *Saccharomycopsis*, *Zygosaccharomyces*, *Schizosaccharomyces*, *Rhizopus*, and *Wickerhamomyces* (Figure 2d), which is consistent with the previous research results [33]. Among them, *Pichia* (STF: 37.44% > WL: 31.78% > NF: 29.56%), *Lichtheimia* (STF: 18.45% > NF: 16.47% > WL: 13.25%), *Monascus* (WL: 18.98% > NF: 14.67% > STF: 8.56%), and *Saccharomyces* (WL: 17.76% > STF: 10.97% > NF: 9.23%) had a combined average relative abundance exceeding 60% throughout the 1–7 day fermentation period, making them the dominant fungal genera in the stacking fermentation of sauce-flavor *Baijiu*. Previous studies have shown that *Pichia* and *Saccharomyces* are the main alcohol-producing functional genera during the fermentation of sauce-flavor *Baijiu* [22,34]. In abnormal fermentation, the combined relative abundance of *Pichia* and *Saccharomyces* was higher than in normal fermentation (WL: 48.23% > STF: 47.67% > NF: 38.56%). The high relative abundance of *Pichia* and *Saccharomyces* inevitably led to high ethanol concentrations in the fermentation environment, inhibiting the growth, reproduction, and metabolism of other microorganisms, and resulting in a simplified microbial community structure, causing abnormal fermentation [35]. Additionally, *Zygosaccharomyces*, which has strong tolerance and can significantly enhance various aromatic components in sauce-flavor *Baijiu* [36], had a higher proportion in normal fermentation (Figure 2e).

### 3.3. Differences in Microbial Community Assembly Mechanisms

**Figure 3:**
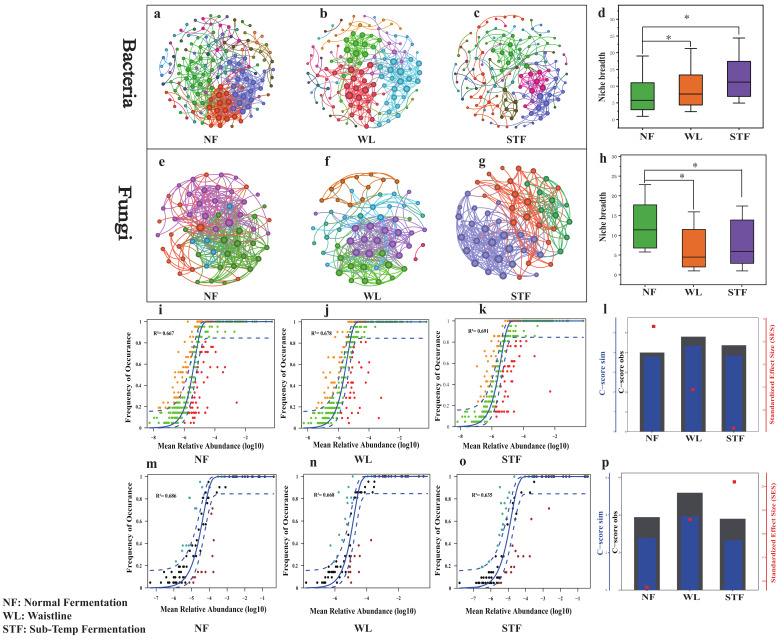
Microbial co-occurrence network analysis: co-occurrence network of bacterial community for (**a**) normal fermentation, (**b**) Waistline, and (**c**) Sub-Temp Fermentation. Co-occurrence network of fungal community for (**e**) normal fermentation, (**f**) Waistline, and (**g**) Sub-Temp Fermentation. Analysis of microbial community assembly mechanism: neutral community model of bacterial community for (**i**) normal fermentation, (**j**) Waistline, and (**k**) Sub-Temp Fermentation. Neutral community model of fungal community for (**m**) normal fermentation, (**n**) Waistline, and (**o**) Sub-Temp Fermentation. C-score score plots for bacterial (**l**) and fungal (**p**) communities. Niche widths for bacterial (**d**) and fungal (**h**) communities. The * indicate statistical significance at *p* < 0.05. Different colours in the network diagram represent different modules. Green in the box-and-line diagram represents NF; orange represents WL; and purple represents STF samples. The blue colour in the bar chart corresponds to the C-Score sim value; black represents the C-Score obs value; and red represents the SES value. green and black represent Neutral, orange and blue represent Above, and red and burgundy represent Below in the NCM Neutral Community Model.

In normal fermentation (Figure 3a,e), the microbial community structure was complex and highly interconnected, which contributed to fermentation stability. In contrast, in Waistline (Figure 3b,f) and Sub-Temp Fermentation (Figure 3c,g) states, the microbial community structure was simplified, dispersed, and showed significant differentiation, indicating potential metabolic blockages in the community. The network topology properties table (Table S1) for different fermentation states show that in normal fermentation, both bacterial and fungal networks had higher numbers of nodes (Bacteria-NF: 151 > STF: 134 > WL: 128, Fungi-NF: 67 > STF: 63 > NF: 56) and edges (Bacteria-NF: 496 > WL: 349 > STF: 231, Fungi-NF: 338 > WL: 293 > STF: 254). This indicates that the microbial community in normal fermentation was richer and more complex. This complexity was reflected in higher biological α-diversity (Figure S4) and higher local interconnectedness (such as high average clustering coefficient and transitivity) (Table S1). These characteristics helped form a stable ecological network and maintain fermentation stability. In contrast, the networks in Waistline and Sub-Temp Fermentation states had fewer nodes and edges, reflecting the simplification and reduced functionality of the microbial communities in abnormal fermentation.

Using NCM models, niche breadth, and C-score methods to jointly assess the relative importance of deterministic and stochastic processes in microbial community assembly under different fermentation states [27], the R^2^ values of bacterial communities in the two types of abnormal fermentation (Figure 3j,k) were higher than those in normal fermentation (Figure 3i) (STF: 0.691 > WL: 0.678 > NF: 0.667). This indicates that the bacterial community assembly in abnormal fermentation was more influenced by stochastic events, likely due to factors such as sudden temperature drops during winter production, leading to frequent dynamic changes in bacterial communities and increased species turnover and extinction events [37]. Additionally, niche breadth analysis shows that the bacterial communities in normal fermentation, which were more influenced by deterministic factors, had a lower niche breadth (NF: 7.29 < WL: 8.02 < STF: 8.47) (Figure 3d), indicating greater metabolic plasticity and adaptability to environmental changes in the latter [38]. In the C-score analysis, the bacterial community SES values were highest in normal fermentation (NF: 13.81 > WL: 10.87 > STF: 9.27) (Figure 3l), indicating that the bacterial community structure in normal fermentation was strongly influenced by ecological driving factors and competitive interactions. The lower SES values in the bacterial communities of abnormal fermentation suggest a structure closer to a random model, more susceptible to environmental fluctuations, leading to fermentation instability [39]. In contrast, the fungal communities in abnormal fermentation (Figure 3n,o) had lower R2 values (NF: 0.686 > WL: 0.668 > STF: 0.635), higher niche breadth (Figure 3h), and higher SES values (Figure 3p) compared to normal fermentation. This implies that the instability of the abnormal fermentation environment forces fungi to dominate resource competition through complex interactions and adaptation mechanisms [8,40], thus being more influenced by deterministic factors. Conversely, bacteria respond more quickly to environmental fluctuations and have more fixed metabolic processes, making them more influenced by stochastic events [41].

### 3.4. Screening of Microbial Biomarkers Based on Machine Learning

**Figure 4:**
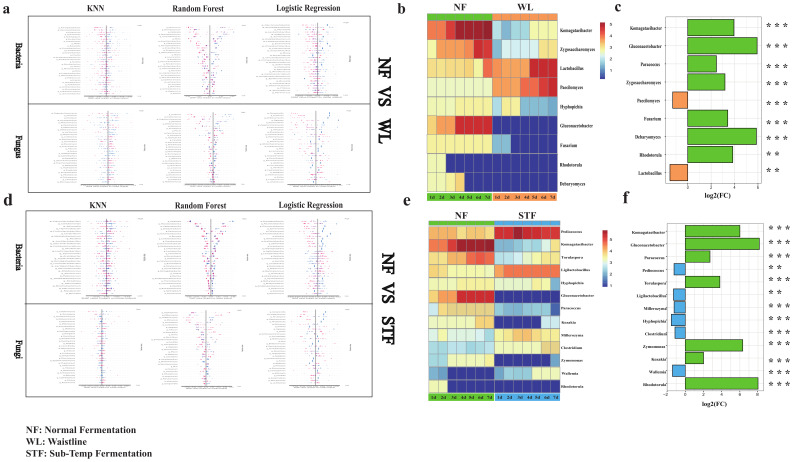
Plots of feature importance ranking of the three machine learning SHAP models: (**a**) normal fermentation vs. Waistline and (**d**) normal fermentation vs. Sub-Temp Fermentation. Heatmap of microbial marker relative abundance dynamics during stacking fermentation: (**b**) Waistline vs. normal fermentation and (**e**) Sub-Temp Fermentation vs. normal fermentation. Histogram of fold change in microbial biomarkers: (**c**) Waistline vs. normal fermentation and (**f**) Sub-Temp Fermentation vs. normal fermentation. The ** and *** indicate statistical significance at *p* < 0.01, and *p* < 0.001, respectively. *p* < 0.05 and |log_2_Fc| > 1 were considered significant. The green colour in the figure represents the NF sample, the orange colour represents the WL sample and the blue colour represents the STF sample.

Using Random Forest, Logistic Regression, and KNN machine learning algorithms, we constructed diagnostic prediction models for abnormal stacking fermentation in sauce-flavor *Baijiu*, achieving good model evaluations. Three algorithms demonstrated strong classification performance across tasks, with high AUC values, such as 1.0 (Random Forest), 0.94 (Logistic Regression), and 0.86 (KNN) in NF vs. STF (Bacteria). The F1-Scores were similarly robust, reaching 0.89, 0.91, and 0.74 for Random Forest, Logistic Regression, and KNN, respectively, in NF vs. STF (Fungi). Accuracy remained consistent, such as 0.85, 0.90, and 0.90 in NF vs. WL (Fungi), while Recall (e.g., 1.0 for Random Forest) and Precision (e.g., 0.93 for Logistic Regression) validated reliability. These results confirm the robustness and applicability of all three models (Table S2). The SHAP method was used to interpret the model feature rankings (Figure 4a,d). Nine biomarkers were identified for Waistline, including *Komagataeibacter* (*p* < 0.001, log_2_Fc = 3.95), *Gluconacetobacter* (*p* < 0.001, log_2_Fc = 5.93), *Paecilomyces* (*p* < 0.001, log_2_Fc = −1.28), Lactobacillus (*p* < 0.01, log_2_Fc = −1.51), etc. (Figure 4b). Thirteen microbial biomarkers were identified for Sub-Temp Fermentation, including *Komagataeibacte*r (*p* < 0.001, log_2_Fc = 6.01), *Gluconacetobacter* (*p* < 0.001, log_2_Fc = 8.14), Pediococcus (*p* < 0.001, log_2_Fc = −1.23), Ligilactobacillus (*p* < 0.001, log_2_Fc = −1.29), etc. (Figure 4e). Notably, the relative abundances of *Komagataeibacter* and *Gluconacetobacter* showed significant differences between normal fermentation and the two types of abnormal fermentation (*p* < 0.0001, log2Fc > 1) (Figure 4c,f). Previous studies have shown that *Komagataeibacter* and *Gluconacetobacter* are high producers of bacterial cellulose [42], which provides a protective barrier for microorganisms in the fermentation system [43], protecting against external changes and harmful substances and improving the quality of fermented foods [44,45]. Additionally, during the 1–7 day stacking fermentation period, the relative abundance of *Lactobacillus* in Waistline was significantly higher than in normal fermentation (Figure 4b), and the relative abundance of *Ligilactobacillus* in Sub-Temp Fermentation was significantly higher than in normal fermentation (Figure 4e). The previous literature has shown that *Lactobacillus* and *Ligilactobacillus* have high lactic acid production capacity, which may explain the significantly higher lactic acid content in abnormal fermentation compared to normal fermentation (NF: 3.96 ± 0.7 mg/g < STF: 4.51 ± 0.7 mg/g < WL: 4.87 ± 1.3 mg/g) (Figure S3d). However, non-volatile acids, like lactic acid, can accumulate during the fermentation process of sauce-flavor *Baijiu*, leading to excessively high lactic acid levels, which inhibit the growth, reproduction, and metabolism of microorganisms [46], thereby affecting fermentation stability and the quality of *Baijiu*.

### 3.5. Analysis of Volatile Compound Differences and Screening of Flavor Biomarkers

**Figure 5:**
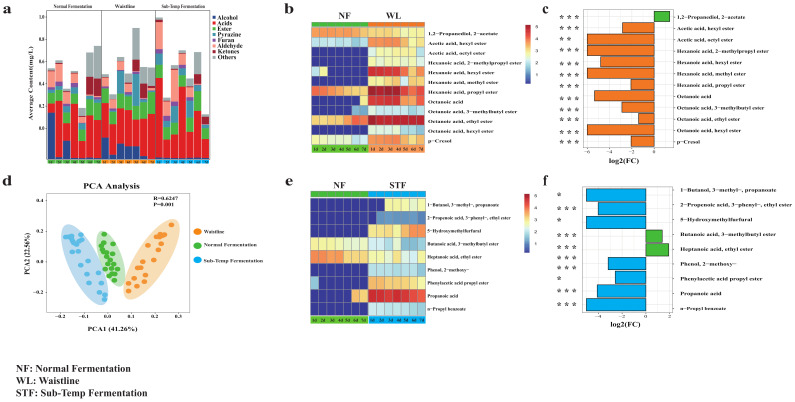
(**a**) Average concentration content of volatile components during stacking fermentation. Heatmap of the dynamic change of flavor marker concentration during stacking fermentation: (**b**) normal fermentation vs. Waistline and (**e**) normal fermentation vs. Sub-Temp Fermentation. Histogram of flavor marker fold change: (**c**) normal fermentation vs. Waistline and (**f**) normal fermentation vs. Sub-Temp Fermentation. (**d**) Principal component analysis of volatile components. The *, **, and *** indicate statistical significance at *p* < 0.05, *p* < 0.01, and *p* < 0.001, respectively. *p* < 0.05 and |log_2_Fc| > 1 were considered significant.

We used HS-SPME-GCMS to detect the volatile compounds in *Jiupei* during the 1–7 day stacking fermentation period under different fermentation states. The volatile compounds showed significant differences (*p* < 0.05) in their concentrations across different fermentation states. The principal component analysis (PCA) of the volatile compounds explained 63.82% of the total variance, with the first two principal components accounting for 63.82% of the variation (Figure 5d). PCA was performed on all detected volatile compounds, and the results were based on those compounds exhibiting significant variation (*p* < 0.05) across fermentation states. A total of 173 volatile compounds were detected in normal fermentation, 179 in Sub-Temp Fermentation, and 198 in Waistline (Figure S5i,j,k). This was consistent with the previous findings of Cao et al. [20], where the number of volatile compounds in medium- and low-quality Daqu was higher than in high-quality Daqu. Acid and ester compounds were dominant during the 1–7 day fermentation period and showed significant differences in concentration (*p* < 0.05) (Figure S5b,c). The concentration of ester compounds was higher in both types of abnormal fermentation compared to normal fermentation (WL: 0.16 ± 0.05 mg/L > STF: 0.12 ± 0.02 mg/L > NF: 0.06 ± 0.02 mg/L), and the concentration of acid compounds was also higher in abnormal fermentation (STF: 0.36 ± 0.22 mg/L > WL: 0.31 ± 0.02 mg/L > NF: 0.25 ± 0.16 mg/L) (Figure 5a).

To better reveal the differences in volatile compounds under different fermentation states, we applied a machine learning-based biomarker screening process to select volatile compounds, achieving good model evaluations (Figure S5l) (Table S3). We identified nine biomarkers for Sub-Temp Fermentation, including propionic acid (*p* < 0.0001, log_2_Fc = −4.0917), ethyl heptanoate (*p* < 0.0001, log_2_Fc = 1.91), 5-hydroxymethylfurfural (*p* < 0.05, log_2_Fc = −4.54), etc. (Figure 5e). Twelve biomarkers were identified for Waistline, including octanoic acid (*p* < 0.001, log_2_Fc = −5.34), hexyl hexanoate (*p* < 0.01, log_2_FC = −4.79), p-cresol (*p* < 0.001, log_2_Fc = −2.06), etc. (Figure 5b). Overall, ester compounds were the most common type of flavor biomarkers, and their concentrations in abnormal fermentation were significantly higher than in normal fermentation (*p* < 0.001, log_2_FC < −1). Previous studies have indicated that ester and acid compounds are important aroma substances in the fermentation of *Baijiu*. However, excessive concentrations of acids and esters can disrupt the aroma balance, leading to off-flavors [47,48]. For instance, high concentrations of octanoic acid can produce sweaty and fatty odors, while propionic acid can produce unpleasant, rancid, and pungent smells [49]. Ester compounds, like ethyl hexanoate and ethyl heptanoate, can generate unpleasant odors [48]. Therefore, we suggest that the off-flavor characteristics of *Jiupei* in abnormal fermentation might be related to the high content of acid and ester volatile compounds (Figure 5b,e). In addition to acid and ester compounds, the Waistline biomarkers included p-cresol, which had a muddy odor (Figure 5b) [50], and 5-hydroxymethylfurfural, which caused bitterness in *Baijiu*, was identified as a Sub-Temp Fermentation biomarker (Figure 5e) [51].

### 3.6. Correlation Analysis of Biomarkers and Environmental Driving Factors

**Figure 6:**
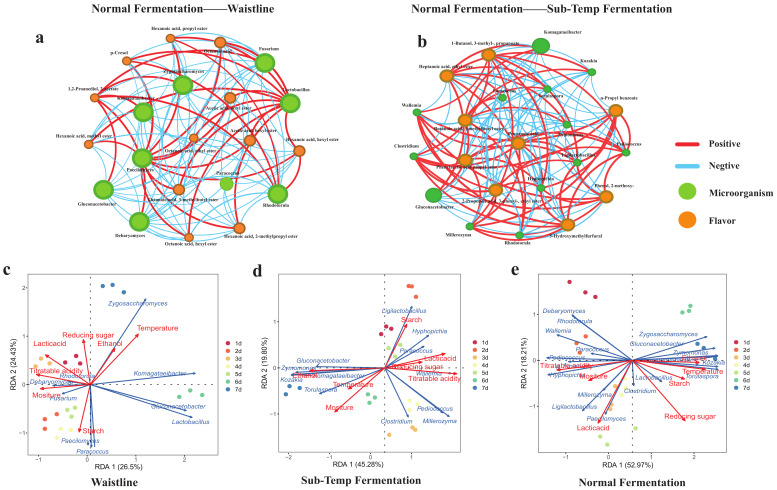
Spearman correlation network analysis of microbial markers with flavor markers: (**a**) normal fermentation vs. Waistline and (**b**) normal fermentation vs. Sub-Temp Fermentation. The positive edges (Spearman’s ρ > 0.6) are represented in red, and the negative edges (Spearman’s ρ < −0.6) are represented in blue. RDA analysis: (**c**) Waistline, (**d**) Sub-Temp Fermentation, and (**e**) normal fermentation. The dotted lines in the RDA diagram represent the axes.

Based on the selected microbial and flavor biomarkers, Spearman correlation analysis was conducted. A total of 102 pairs of Waistline microbial biomarkers and volatile compounds were found to be correlated, with 31 pairs showing positive correlations and 71 pairs showing negative correlations (Figure 6a). Among them, *Gluconacetobacter* and *Komagataeibacter* were mostly negatively correlated with acid and ester flavor biomarkers in Waistline. For example, ethyl hexanoate was negatively correlated with *Gluconacetobacter* (ρ = −0.87, *p* < 0.05) and *Komagataeibacter* (ρ = −0.86, *p* < 0.05), which might be one reason for the lower ester concentrations in normal fermentation compared to abnormal fermentation. The correlations between microbial biomarkers and flavor biomarkers in Sub-Temp Fermentation were more balanced, with 117 pairs of microbial biomarkers and volatile compounds identified, including 56 positive correlations and 61 negative correlations (Figure 6b). For instance, propionic acid, which had a pungent and sour taste, was significantly negatively correlated with *Gluconacetobacter* (ρ = −0.74, *p* < 0.05) and *Komagataeibacter* (ρ = 0.71, *p* < 0.05). This study further indicates that *Gluconacetobacter* and *Komagataeibacter* were key functional genera for maintaining normal fermentation.

Environmental factors significantly drove normal fermentation (F = 5.022, *p* < 0.001), with RDA explaining 71.18% of the variance. Reducing sugars (r^2^ = 0.88, *p* = 0.001), titratable acidity (r^2^ = 0.82, *p* = 0.001), temperature (r^2^ = 0.79, *p* = 0.001), ethanol (r^2^ = 0.65, *p* = 0.001), and lactic acid (r^2^ = 0.61, *p* = 0.001) significantly co-regulated fermentation (Figure 6e). The synergistic regulation of multiple environmental factors favored the production of bacterial cellulose by *Gluconacetobacter* and *Komagataeibacter* biomarkers, thereby maintaining normal fermentation. This was consistent with previous studies indicating that bacterial cellulose synthesis pathways are influenced by reducing sugars, moderate ethanol, and lactic acid [52]. Environmental factors also significantly drove Sub-Temp Fermentation (F = 3.56, *p* < 0.001), with RDA explaining 65.08% of the variance. Ethanol (r^2^ = 0.85, *p* = 0.002), titratable acidity (r^2^ = 0.78, *p* = 0.001), and moisture (r^2^ = 0.67, *p* = 0.002) significantly regulated Sub-Temp Fermentation (Figure 6d). Comparing the RDA analysis results with normal fermentation, we identified moisture as a significant driving factor for the differences in Sub-Temp Fermentation. The lack of co-regulation by reducing sugars, lactic acid, and temperature indirectly led to Sub-Temp Fermentation. Additionally, maintaining a low moisture state throughout the 1–7 day fermentation process (Figure 1b) suggested that adding moisture during production could improve the regulation of Sub-Temp Fermentation. The environmental factor regulation of Waistline was significantly different from the other two fermentation types (*p* = 0.01, F = 1.96), with RDA explaining only 50.93% of the variance. The correlations with temperature, lactic acid, and titratable acidity were relatively low (r^2^ < 0.6) (Figure 6c). This could be due to the dominance of bacterial biomarkers in Waistline (Figure 4b) and the bacterial community assembly in abnormal fermentation being more influenced by stochastic ecological processes.

### 3.7. Differential Gene Pathway Enrichment and Predicted Metabolic Network Analysis for Characteristic Flavor Formation

**Figure 7:**
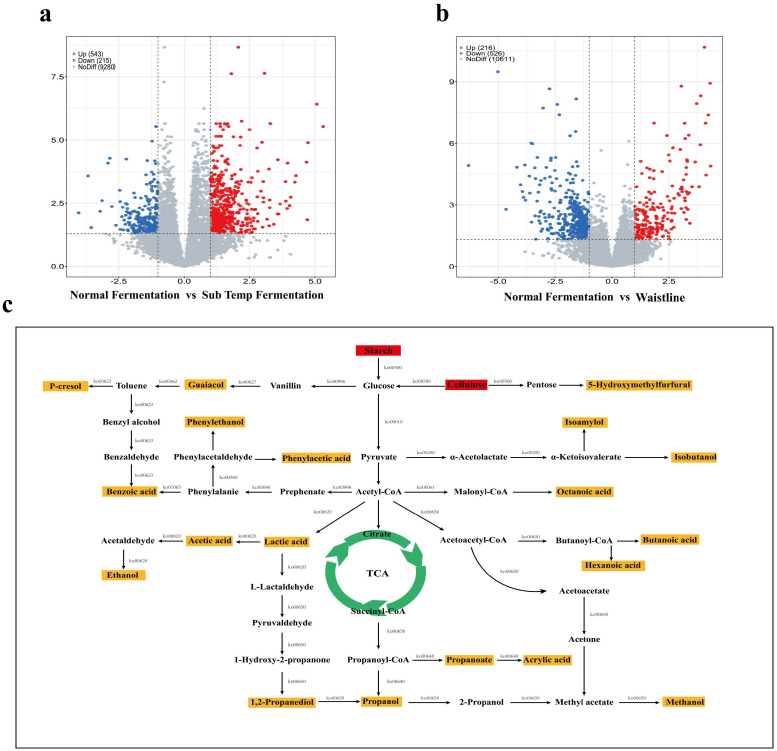
DESqe2 differential gene volcano map: (**a**) normal fermentation vs. Sub−Temp Fermentation and (**b**) normal fermentation vs. Waistline. (**c**) Flavor marker metabolic pathway network preiction based on KEGG data. The dotted lines in the volcano map represent the axes.

Based on metagenomic data, DESeq2 differential gene analysis identified 758 differentially expressed genes between normal fermentation and Sub-Temp Fermentation, with 543 genes upregulated and 215 genes downregulated (Figure 7a). Between normal fermentation and Waistline, 742 differentially expressed genes were identified, with 216 genes upregulated and 526 genes downregulated (Figure 7b). Pathway enrichment analysis based on these differentially expressed genes using the KEGG database (Figure S6b,c) shows that 71 metabolism-related pathways were enriched for 742 genes between normal fermentation and Waistline, and 42 metabolism-related pathways were enriched for 758 genes between normal fermentation and Sub-Temp Fermentation. Additionally, the metabolic pathways responsible for the formation of flavor biomarkers in abnormal fermentation were predicted (Figure 7c), and the enzyme-encoding genes potentially involved in the production of these flavor biomarkers were identified (Figure S6a).

In abnormal fermentation, the 5-hydroxymethylfurfural responsible for the bitterness in *Baijiu* may be produced via the pentose phosphate pathway from cellulose in the raw materials, which was degraded to pentose through the differential gene-enriched pathway (Figure 7c). Under the high-temperature conditions of stacking fermentation, pentose undergoes the Maillard reaction to form 5-hydroxymethylfurfural [53]. P-cresol, a phenolic compound with a foul odor, may originate from the degradation of ferulic acid and vanillin in the raw materials rather than microbial metabolism during fermentation [54]. Vanillin was decarboxylated by phenacrylate decarboxylase (EC 4.1.1.102) to produce guaiacol, which was then converted to toluene by vanillate monooxygenase (EC 1.14.13.82). In the nitrotoluene degradation pathway, enriched in the differential gene pathway (Figure S6b), toluene was catalytically degraded to p-cresol by arachidonate 8-lipoxygenase (EC 1.13.11.40). For ester compounds, like ethyl acetate, isoamyl octanoate, and isobutyl hexanoate, which are flavor markers in abnormal fermentation, we conducted metabolic pathway analysis using the corresponding acids and alcohols, as KEGG does not provide explicit pathways for ester formation [55]. Organic acids, such as acetic acid, lactic acid, propionic acid, and octanoic acid, primarily originated from starch in the raw materials, which was broken down into glucose by related enzymes. Glucose was further converted into pyruvate through microbial metabolism. Pyruvate was then converted into acetyl-CoA by pyruvate dehydrogenase. Acetyl-CoA was transformed into organic acids through differential gene-enriched pathways, such as propionate metabolism, lipoic acid metabolism, and C5-branched dibasic acid metabolism (Figure S6b,c). The formation of higher alcohols, such as isoamyl alcohol and isobutanol, might occur through the differential metabolic pathways of phenylalanine, tyrosine, and tryptophan biosynthesis (Figure S6b,c). This process primarily involves the conversion of pyruvate to α-acetolactate by acetolactate synthase (EC 2.2.1.6), ketol-acid reductoisomerase (EC 1.1.1.86), and dihydroxy-acid dehydratase (EC 4.2.1.9), followed by conversion to α-ketoisovalerate by valine--pyruvate aminotransferase (EC 2.6.1.66), which is then further converted to isoamyl alcohol and isobutanol.

## 4. Conclusions

Based on machine learning combined with multi-omics, nine microbial markers and twelve flavor markers were identified for Waistline, while thirteen microbial markers and nine flavor markers were identified for Sub-Temp Fermentation. *Komagataeibacter* and *Gluconacetobacter* were significantly less abundant in both abnormal fermentations compared to normal, while *Ligilactobacillus* and *Lactobacillus* were significantly more abundant. Excessive acid and ester markers in abnormal fermentations resulted in off-flavors. RDA analysis indicates that moisture significantly drives Sub-Temp Fermentation, while Waistline is less affected by deterministic environmental factors. This research provides new insights for regulating and improving stacking fermentation to ensure the quality and yield of sauce-flavor *Baijiu*.

## Data Availability

The original contributions presented in this study are included in the article/Supplementary Materials. Further inquiries can be directed to the corresponding author.

## Supplementary Materials

The following supporting information can be downloaded at: https://www.mdpi.com/article/10.3390/foods14020245/s1, Figure S1: (a) Sampling points of *Jiupei*: (b) Waistline, (c) normal fermentation, and (d) Sub-Temp Fermentation. Figure S2: Schematic of the machine learning build process. Figure S3: Box plots of differences in average physical–chemical indicators of stacking fermentation: (a) moisture, (b) titratable acidity, (c) temperature, (d) lactic acid, (e) ethanol, (f) reducing sugar, and (g) starch. The *, **, and *** indicate statistical significance at *p* < 0.05, *p* < 0.01, and *p* < 0.001, respectively. Figure S4: Boxplot of the overall difference in the Shannon index of alpha diversity of stacking fermenting organisms: (a) fungi Shannon, (b) bacteria Shannon. The * and ** indicate statistical significance at *p* < 0.05 and *p* < 0.01, respectively. Figure S5: Boxplot of overall differences in volatile components in stacking fermentation: (a) alcohol, (b) acid, (c) ester, (d) pyrazine, (e) furan, (f) aldehyde, (g) ketone, and (h) other. Pie chart of the number of volatile components in stacking fermentation: (i) normal fermentation, (j) Waistline, and (k) Sub-Temp Fermentation. Plots of feature importance ranking of the three machine learning SHAP models (l). Figure S6: (a) Relative abundance of enzymes encoding pathways involved in metabolic prediction of flavor markers during stack fermentation. Differential gene-based enrichment analysis of the KEGG pathway: (b) normal fermentation vs. Sub-Temp Fermentation and (c) normal fermentation vs. Waistline. Table S1: Topological analysis of microbial co-occurrence network graphs. Table S2: Evaluation of machine learning models for microbiological markers. Table S3: Evaluation of machine learning models for flavor markers. Table S4: (a) normal fermentation vs. Waistline and (b) normal fermentation vs. Sub-Temp Fermentation. Histogram of flavor marker fold change.

## Abbreviations

NF, normal fermentation; WL, Waistline; STF, Sub-Temp Fermentation; KNN, K-Nearest Neighbor; NCM, neutral community model; C-score, checkerboard score; SES, standardized effect size; PCA, principal component analysis; Fc: fold change; SHAP, SHapley Additive exPlanations.
