# Supplementary material for: Machine Learning and Multi-Omics Integration to Reveal Biomarkers and Microbial Community Assembly Differences in Abnormal Stacking Fermentation of Sauce-Flavor Baijiu
Source: Foods. 2025 Jan 14;14(2):245. doi: 10.3390/foods14020245 (PMC11765235; doi:10.3390/foods14020245)
Supplement: Supplementary file 1 [file foods-14-00245-s001.zip › supplementary figures caption and tables.pdf]

Supplementary Materials:

Supplementary Figure S1. (a) Sampling points of *Jiupei*: (b) Waistline, (c) Normal Fermentation, (d) Sub-Temp Fermentation.

Supplementary Figure S2. Schematic of the machine learning build process.

Supplementary Figure S3. Box plots of differences in average physicalchemical indicators of Stacking fermentation: (a) Moisture, (b) Titratable acidity, (c) Temperature, (d) Lactic acid, (e) Ethanol, (f) Reducing sugar, (g) Starch. The *, ** and *** indicate statistical significance at $p < 0.05$, $p < 0.01$ and $p < 0.001$, respectively.

Supplementary Figure S4. Boxplot of the overall difference in the Shannon index of alpha diversity of stacking fermenting organisms. (a) fungi Shannon, (b) Bacteria Shannon. The *, ** and *** indicate statistical significance at $p < 0.05$, $p < 0.01$ and $p < 0.001$, respectively.

Supplementary Figure S5. Boxplot of overall differences in volatile components in stacking fermentation: (a) Alcohol, (b) Acid, (c) Ester, (d) Pyrazine , (e) Furan, (f) Aldehyde, (g) Ketone, (h) other. Pie chart of the number of volatile components in Stakcing fermentation: (i) Normal Fermentation, (j) Waistline, (k) Sub-Temp Fermentation. Plots of feature importance ranking of the three machine learning SHAP models(l).

Supplementary Figure S6. (a) Relative abundance of enzymes encoding pathways involved in metabolic prediction of flavor markers during stack fermentation. Differential gene-based enrichment analysis of the KEGG pathway: (b) Normal Fermentation vs Sub-Temp Fermentation, (c) Normal Fermentation vs Waistline.

Table. S1. Topological analysis of microbial co-occurrence network graphs

Category		Total nodes	Total links	Average clustering coefficient (avgCC)	Averagepathdistance(GD)	Transitivity (Trans)	Modularity (fast_greedy)	Centralization of degree (CD)
**Bacteria**	NF	151	496	0.373	4.266	0.574	0.522	0.130
	WL	128	349	0.353	3.747	0.524	0.64	0.091
	STF	134	231	0.249	6.591	0.463	0.714	0.087
**Fungi**	NF	67	338	0.567	2.147	0.499	0.312	0.432
	WL	56	293	0.583	3.042	0.567	0.441	0.210
	STF	63	254	0.373	4.266	0.574	0.522	0.130

Supplementary Table S2. Evaluation of machine learning models for microbiological markers

Group	Model	AUC	F1	Accuracy	Recall	Precision
NFvsSTF(Bacteria)	Random Forest	1.0	0.93	0.94	0.87	1.0
NFvsSTF(Bacteria)	Logistic Regression	0.94	0.88	0.88	0.88	0.91
NFvsSTF(Bacteria)	KNN	0.86	0.79	0.82	0.75	0.85
NFvsSTF(Fungi)	Random Forest	0.97	0.89	0.88	1.0	0.81
NFvsSTF(Fungi)	Logistic Regression	0.98	0.91	0.92	0.92	0.93
NFvsSTF(Fungi)	KNN	0.92	0.74	0.69	0.92	0.63
NFvsWL(Bacteria)	Random Forest	1.0	0.93	0.94	0.87	1.0
NFvsWL(Bacteria)	Logistic Regression	0.77	0.53	0.59	0.59	0.60
NFvsWL(Bacteria)	KNN	0.61	0.58	0.58	0.62	0.55
NFvsWL(Fungi)	Random Forest	1.0	0.82	0.85	0.7	1.0
NFvsWL(Fungi)	Logistic Regression	0.94	0.89	0.90	0.90	0.92
NFvsWL(Fungi)	KNN	0.94	0.90	0.90	0.90	0.90

Supplementary Table S3. Evaluation of machine learning models for flavour markers

Group	Model	AUC	F1	Accuracy	Recall	Precision
NFvsWL	Random Forest	1.0	0.95	0.95	0.95	0.96
NFvsWL	Logistic Regression	1.0	0.95	0.95	0.95	0.95
NFvsWL	KNN	1.0	0.90	0.90	1.0	0.83
NFvsSTF	Random Forest	1.0	1.0	1.0	1.00	1.0
NFvsSTF	Logistic Regression	1.0	1.0	1.0	1.0	1.0
NFvsSTF	KNN	1.0	1.0	1.0	1.0	1.0

Supplementary Table S4 (a) Normal Fermentation vs Waistline, (b) Normal Fermentation vs Sub-Temp Fermentation. Histogram of flavor markers fold change

(a)

Compound	log2(FC)	pVal
1,2-Propanediol, 2-acetate	1.396397892	4.47E-05
Acetic acid, hexyl ester	-2.834732581	0.001738128
Acetic acid, octyl ester	-6	0.018500092
Hexanoic acid, 2-methylpropyl ester	-6	0.001546489
Hexanoic acid, hexyl ester	-4.798204496	0.002047606
Hexanoic acid, methyl ester	-6	0.000405898
Hexanoic acid, propyl ester	-2.07996119	0.005380455
Octanoic acid	-5.347273841	0.001019259
Octanoic acid, 3-methylbutyl ester	-2.890344121	0.00311574
Octanoic acid, ethyl ester	-1.396450066	0.004527671
Octanoic acid, hexyl ester	-6	0.004248012
p-Cresol	-2.067797142	0.000425377

(b)

Compound	log2(FC)	pVal
1-Butanol, 3-methyl-, propanoate	-5	0.032273531
2-Propenoic acid, 3-phenyl-, ethyl ester	-4.011021722	0.000965505
5-Hydroxymethylfurfural	-5	0.032414677
Butanoic acid, 3-methylbutyl ester	1.370815762	0.000312118
Heptanoic acid, ethyl ester	1.912150201	0.000147586
Phenol, 2-methoxy-	-3.170635491	0.001689808
Phenylacetic acid propyl ester	-2.575430224	0.027490515
Propanoic acid	-4.09169853	9.73E-06
n-Propyl benzoate	-5	8.66E-06
