# Supplementary figures and images for: Machine Learning and Multi-Omics Integration to Reveal Biomarkers and Microbial Community Assembly Differences in Abnormal Stacking Fermentation of Sauce-Flavor Baijiu
Source: Foods. 2025 Jan 14;14(2):245. doi: 10.3390/foods14020245 (PMC11765235; doi:10.3390/foods14020245)

**a**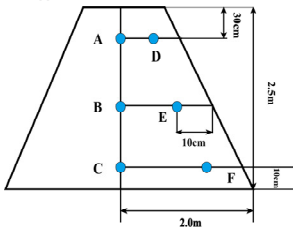**Sampe Diagram**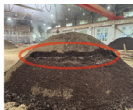**Waistline**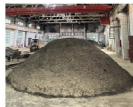**Normal Fermentation**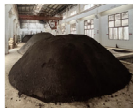**Sub-Temp Fermentation**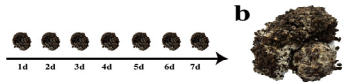**b**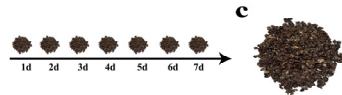**c**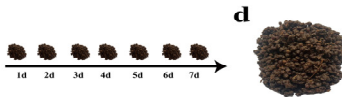**d**

Supplement: Supplementary file 1 [file foods-14-00245-s001.zip › Figure S1.pdf]

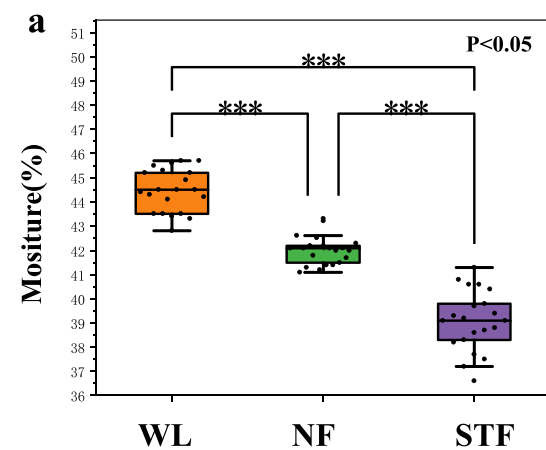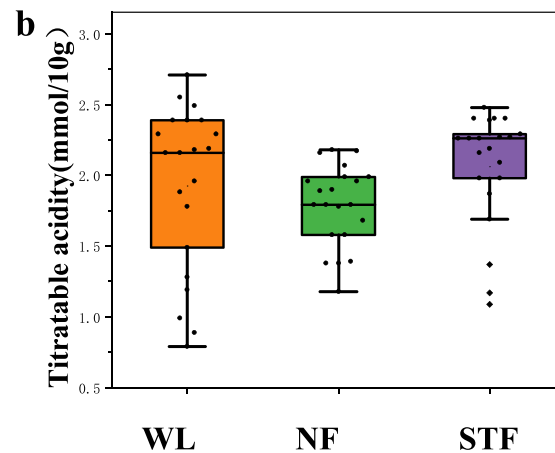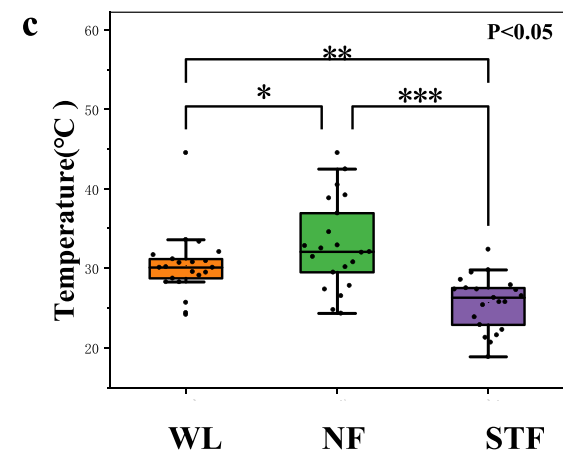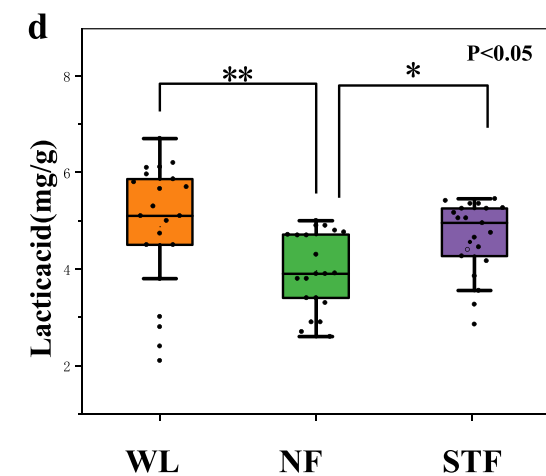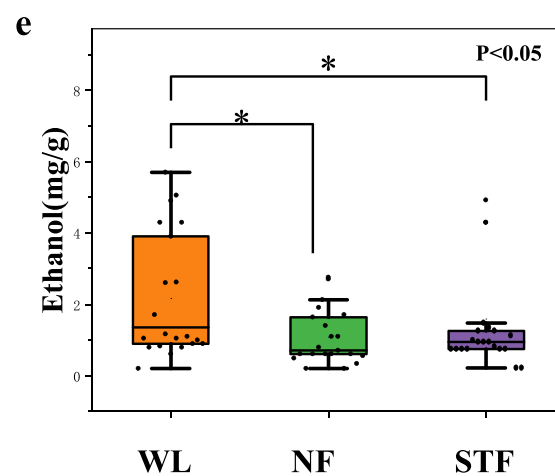

WL: Waistline  
NF: Normal Fermentation  
STF: Sub-Temp Fermentation

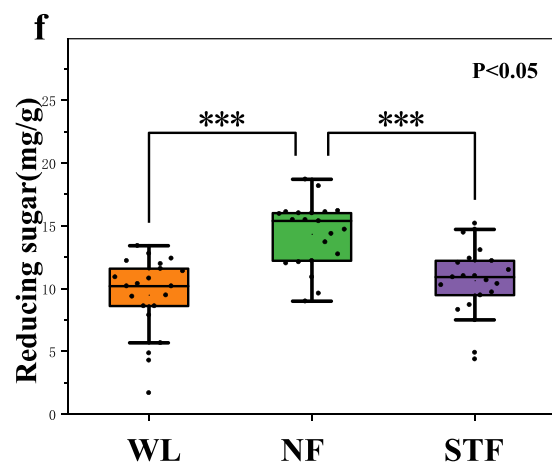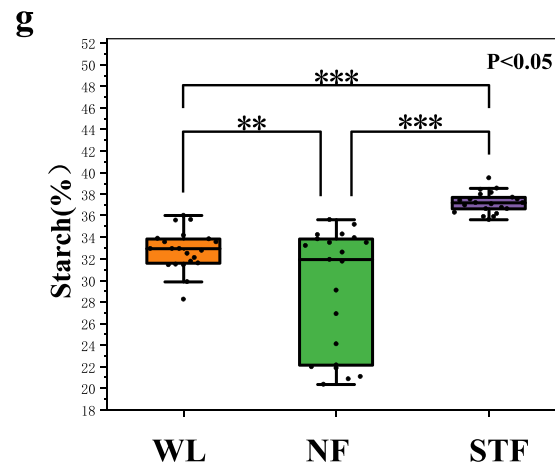

Supplement: Supplementary file 1 [file foods-14-00245-s001.zip › Figure S3.pdf]

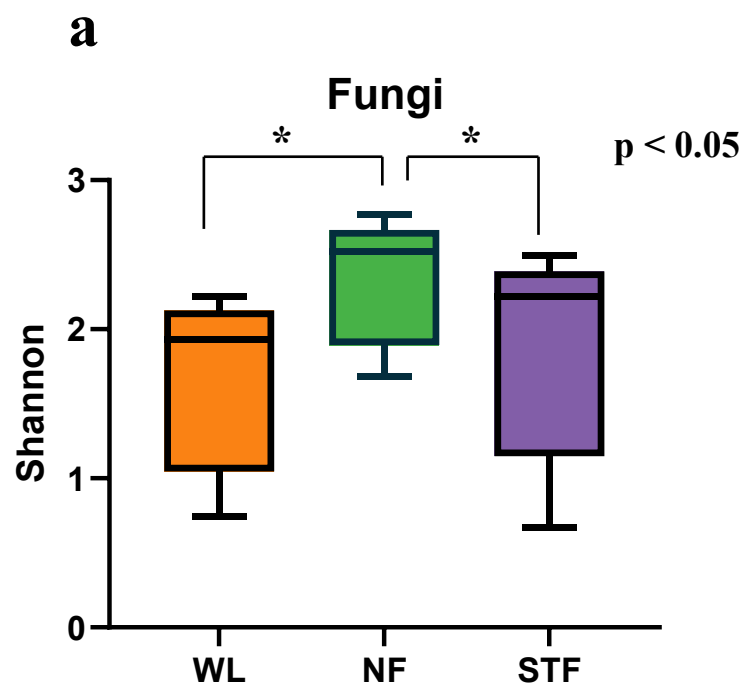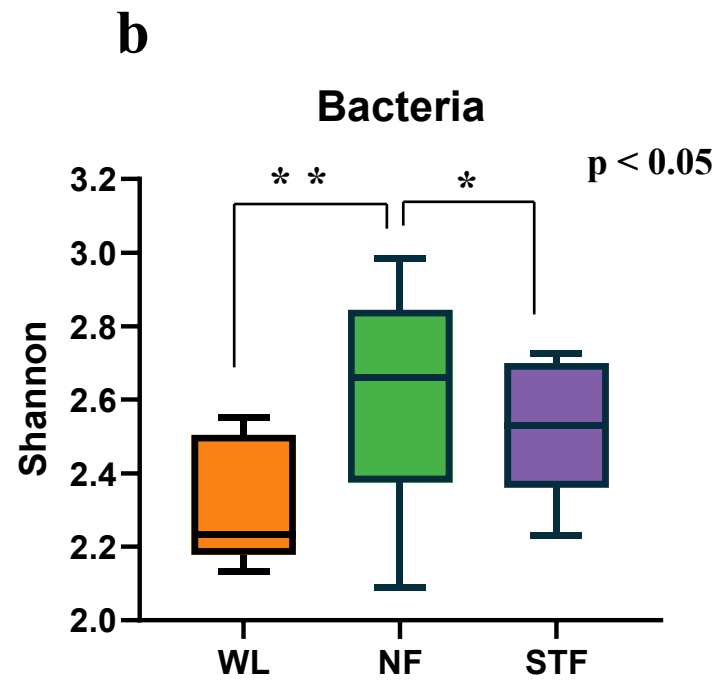

WL: Waistline  
NF: Normal Fermentation  
STF: Sub-Temp Fermentation

Supplement: Supplementary file 1 [file foods-14-00245-s001.zip › Figure S4.pdf]

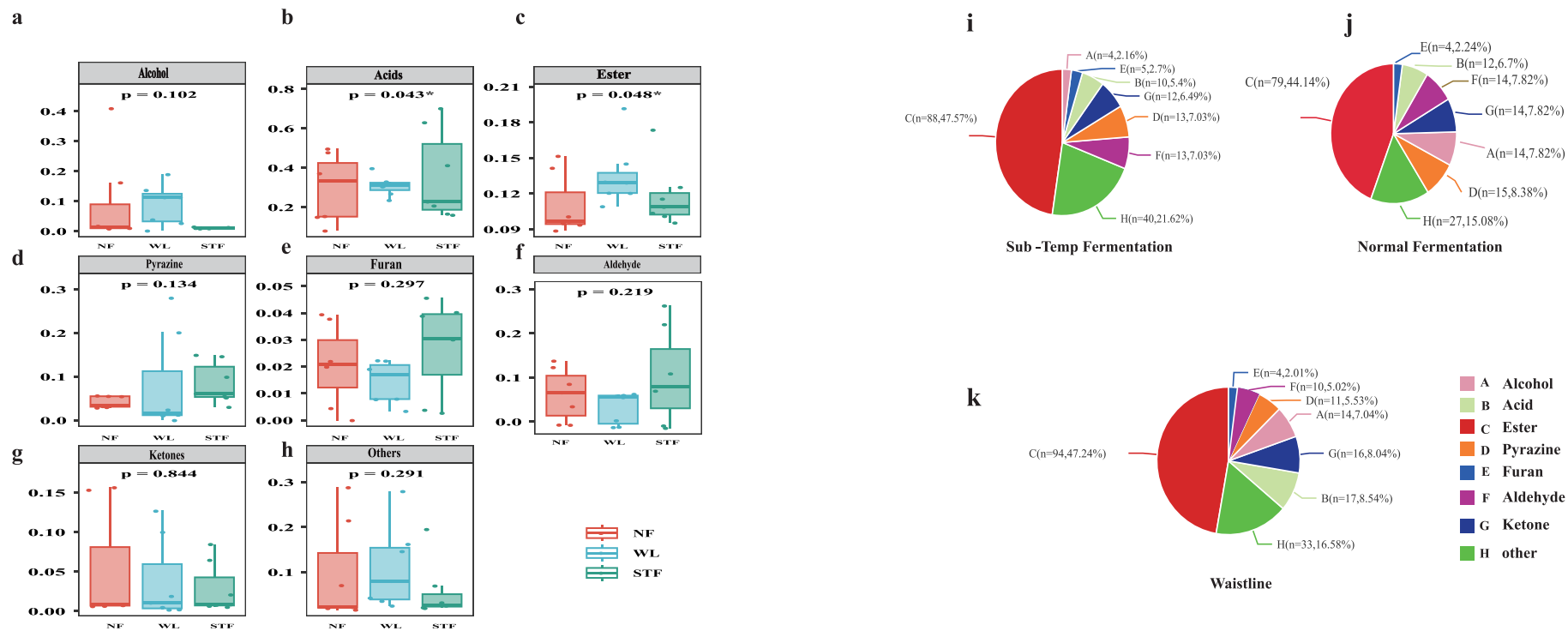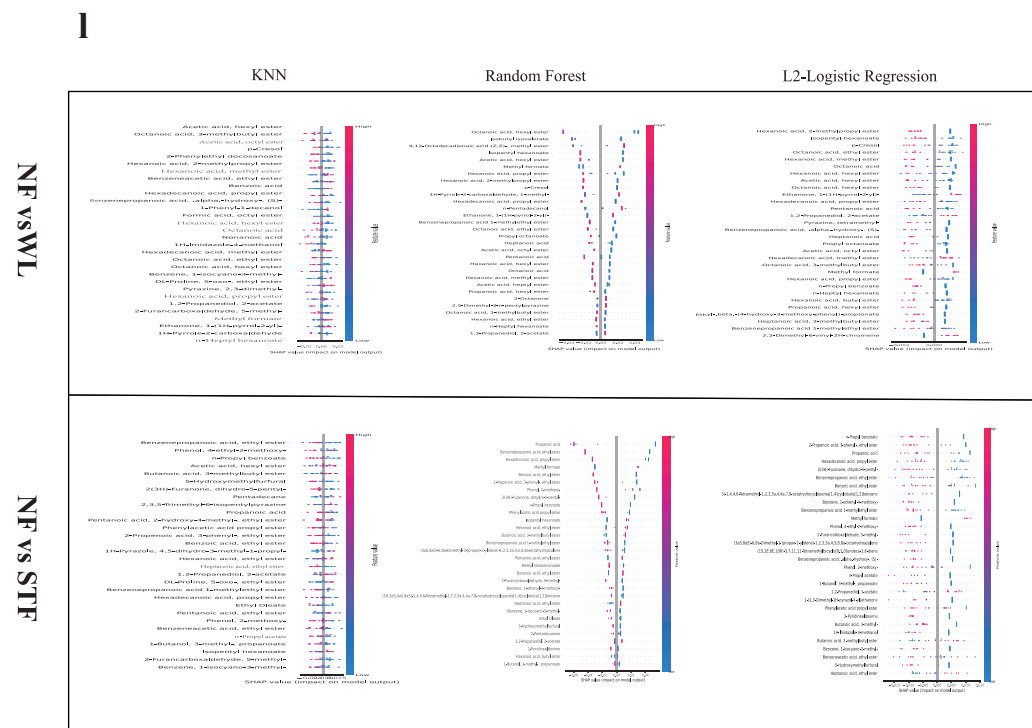

Supplement: Supplementary file 1 [file foods-14-00245-s001.zip › Figure S5.pdf]

a

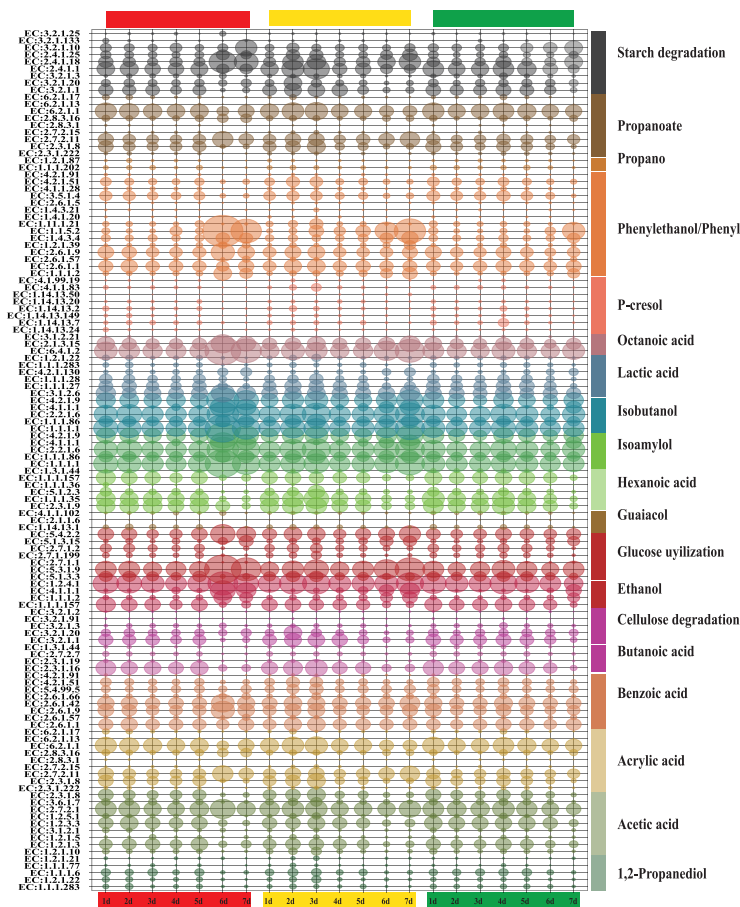

b

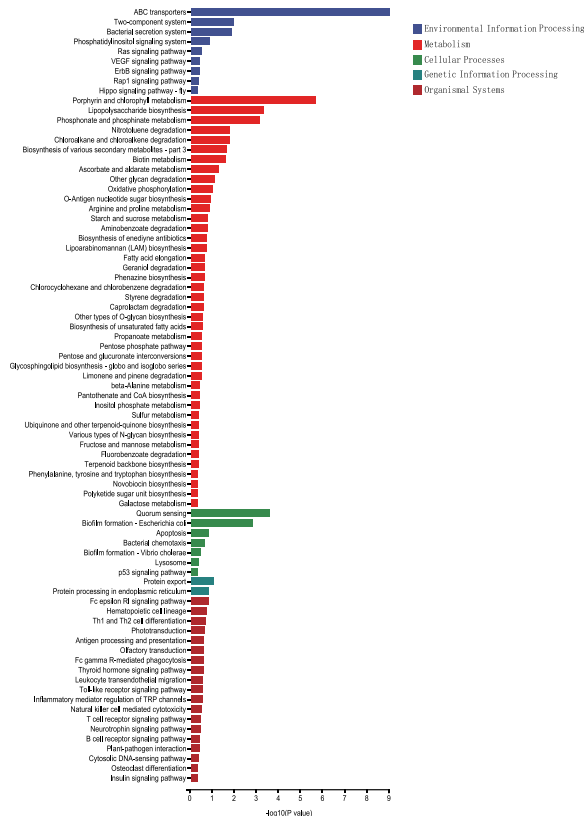

Normal Fermentation vs Sub-Temp Fermentation

c

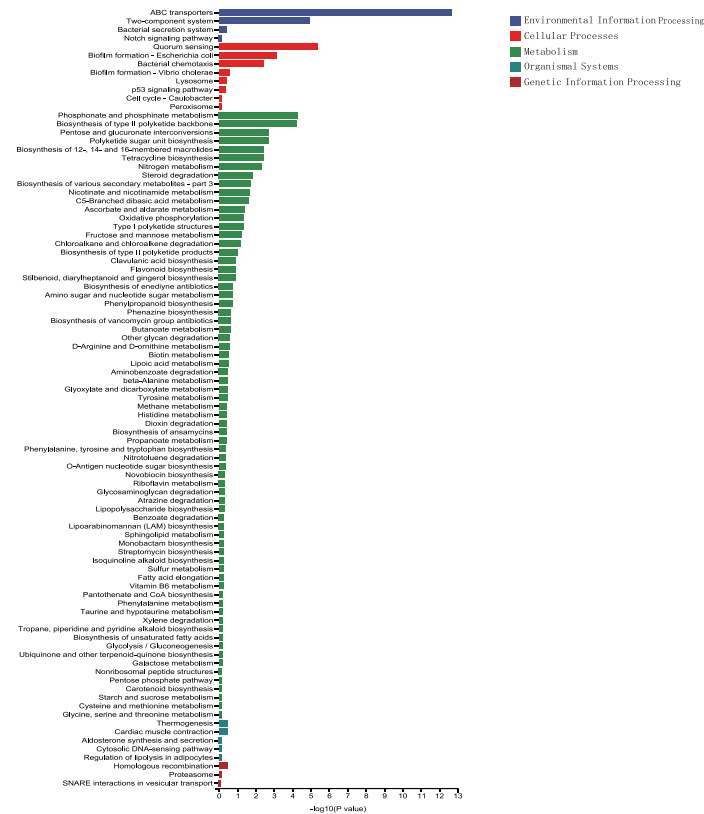

Normal Fermentation vs Waistline

Supplement: Supplementary file 1 [file foods-14-00245-s001.zip › Figure S6.pdf]
