# Supplementary material for: Machine Learning and Multi-Omics Integration to Reveal Biomarkers and Microbial Community Assembly Differences in Abnormal Stacking Fermentation of Sauce-Flavor Baijiu
Source: Foods. 2025 Jan 14;14(2):245. doi: 10.3390/foods14020245 (PMC11765235; doi:10.3390/foods14020245)
Supplement: Supplementary file 1 [file Figure S2.pdf › Figure S2.pdf]

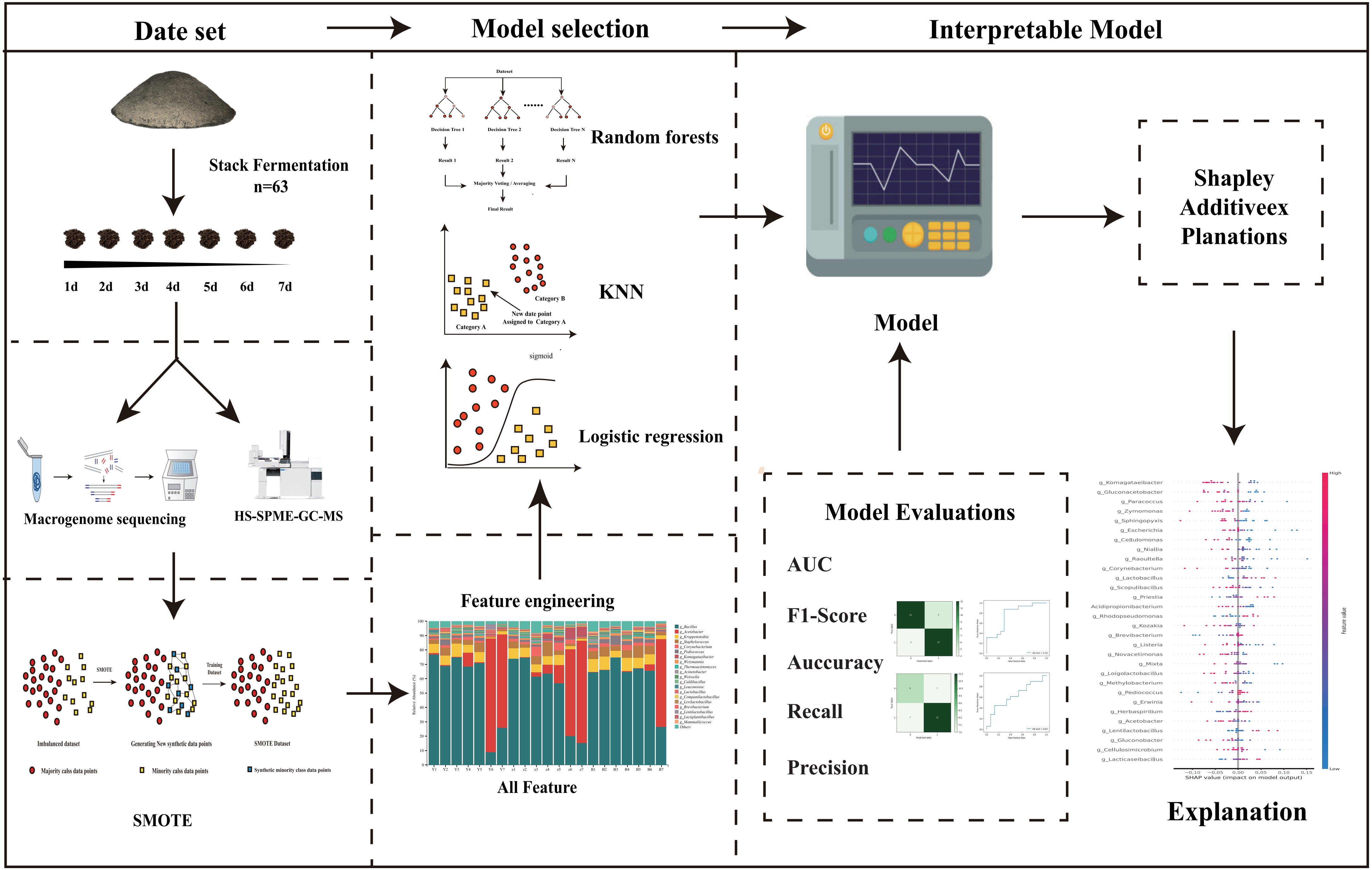
Date set
Model selection
Interpretable Model
Stack Fermentation
n=63
1d
2d
3d
4d
5d
6d
7d
Macrogenome sequencing
HS-SPME-GC-MS
SMOTE
Training Dataset
Imbalanced dataset
Generating New synthetic data points
SMOTE Dataset
Majority calss data points
Minority calss data points
Synthetic minority class data points
SMOTE
Dateset
Decision Tree 1
Decision Tree 2
Decision Tree N
Result 1
Result 2
Result N
Majority Voting / Averaging
Final Result
Random forests
Category B
New date point
Assigned to Category A
Category A
KNN
sigmoid
Logistic regression
Feature engineering
All Feature
Model
Shapley
Additiveex
Planations
Model Evaluations
AUC
F1-Score
Auccuracy
Recall
Precision
SHAP value (impact on model output)
Feature value
High
Low
Explanation
